# Supplementary figures and images for: Antibody responses against SARS-CoV-2 variants induced by four different SARS-CoV-2 vaccines in health care workers in the Netherlands: A prospective cohort study
Source: PLoS Med. 2022 May 17;19(5):e1003991. doi: 10.1371/journal.pmed.1003991 (PMC9113667; doi:10.1371/journal.pmed.1003991)

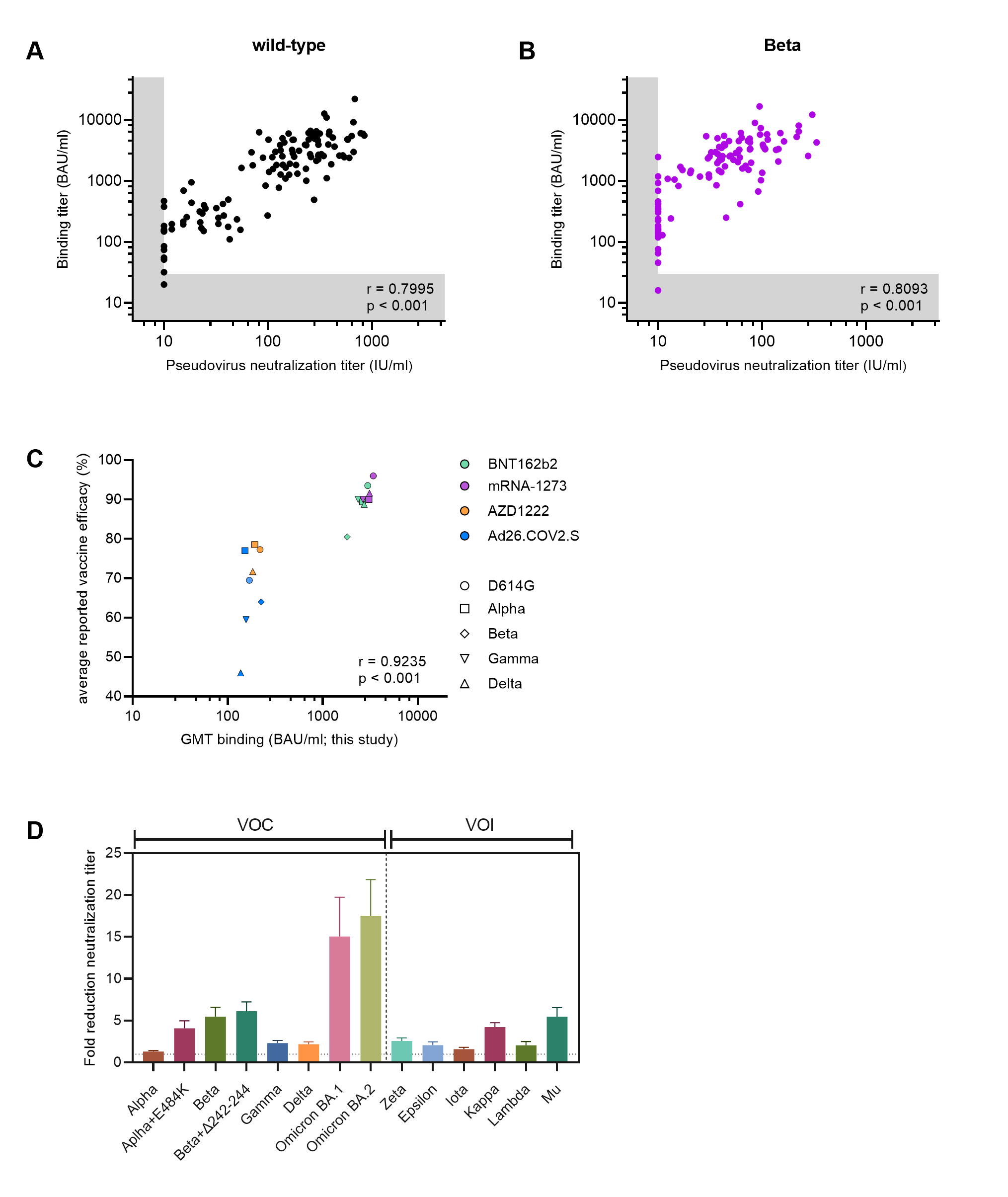

Supplement: S3 Fig — Correlation between wild-type (A) and Beta (B) binding (BAU/ml) and neutralization half-maximal inhibitory concentration (IC50) titers (IU/ml). Spearman’s rank correlation coefficient with p-value are indicated. (C) Geometric mean titers (GMT; BAU/ml) of wild-type and VOCs plotted against the average reported vaccine efficacy against symptomatic infection with WT or VOCs (S2 and S4 Tables). Vaccine groups are indicated by colors with BNT162b2 in green, mRNA-1273 in purple, AZD1222 in orange and Ad26.COV2.S in blue. Circles represent WT data, squares for Alpha, diamond for Beta, nabla triangle for Gamma and delta triangle for Delta. Spearman’s rank correlation coefficient with p-value are indicated. (D) Mean ± SEM fold reductions in neutralization IC50 titers for the serum pools combined against the VOCs and VOIs pseudoviruses in comparison to IC50 titers against D614G pseudovirus. (TIF) [file pmed.1003991.s004.tif]
