# Supplementary material for: Antibody responses against SARS-CoV-2 variants induced by four different SARS-CoV-2 vaccines in health care workers in the Netherlands: A prospective cohort study
Source: PLoS Med. 2022 May 17;19(5):e1003991. doi: 10.1371/journal.pmed.1003991 (PMC9113667; doi:10.1371/journal.pmed.1003991)
Supplement: S1 Table — (DOCX) [file pmed.1003991.s005.docx]

**Table S1: Protein and pseudovirus S constructs contain the following mutations compared to the WT (Wuhan Hu-1; GenBank: MN908947.3).**

| **VOC^#^ S proteins** | | | | | | | | | | | | | | | |  |  |
| --- | --- | --- | --- | --- | --- | --- | --- | --- | --- | --- | --- | --- | --- | --- | --- | --- | --- |
| **Alpha** | | **Beta** | | **Gamma** | | | **Beta** | | | **Omicron** | | | | | |  |  |
| B.1.1.7 | | B.1.351 | | P.1 | | | B.1.351 | | | B.1.1.529 | | | | | |  |  |
| Δ69-70 | | L18F | | L18F | | | T19R | | | A67V | | S375F | Y505H | | |  |  |
| Δ144 | | D80A | | T20N | | | G142D | | | Δ69-70 | | K417N | T547K | | |  |  |
| N501Y | | D215G | | P26S | | | E156G | | | T95I | | N440K | D614G | | |  |  |
| A570D | | Δ242-244 | | D138Y | | | Δ157-158 | | | G142D | | G446S | H655Y | | |  |  |
| D614G | | K417N | | R190S | | | L452R | | | Δ143-145 | | S477N | N679K | | |  |  |
| P681H | | E484K | | K417T | | | T478K | | | Δ211 | | T478K | P681H | | |  |  |
| T716I | | N501Y | | E484K | | | D614G | | | L212I | | E484A | N764K | | |  |  |
| S982A | | D614G | | N501Y | | | P681R | | | ins214EPE | | Q493K | D796Y | | |  |  |
| D1118H | | A701V | | D614G | | | D950N | | | G339D | | G496S | N856K | | |  |  |
|  | |  | | H655Y | | |  | | | S371L | | Q498R | Q954H | | |  |  |
|  | |  | | T1027I | | |  | | | S373P | | N501Y |  | | |  |  |
|  | |  |  | | |  | | |  | | | | |  |  |  |  |
|  | |  |  | | |  | | |  | | | | |  |  |  |  |
|  | **VOC^#^ pseudovirus** | | | | | | | | | | | | | | | | |
| **D614G** | **Alpha** | | **Alpha** | | **Beta** | | | **Beta** | | | **Gamma** | **Delta** | | | **Omicron** | | **Omicron** |
|  |  |  | **+E484K** | |  |  |  | **+242-244** | | |  |  |  |  |  |  |  |
| B.1 | B.1.1.7 | | B.1.1.7 | | B.1.351 | | | B.1.351 | | | P.1 | B.1.617.2 | | | B.1.1.529 BA.1 | | B.1.1.529 BA.2 |
| D614G | Δ69-70 | | Δ69-70 | | L18F | | | L18F | | | L18F | T19R | | | A67V | | T19I |
|  | Δ144 | | Δ144 | | D80A | | | D80A | | | T20N | G142D | | | Δ69-70 | | L24S |
|  | N501Y | | E484K | | D215G | | | D215G | | | P26S | E156G | | | T95I | | Δ125/127 |
|  | A570D | | N501Y | | Δ242-244 | | | L242H | | | D138Y | Δ157-158 | | | G142D | | G142D |
|  | D614G | | A570D | | K417N | | | R246I | | | R190S | L452R | | | Δ143-145 | | V213G |
|  | P681H | | D614G | | E484K | | | K417N | | | K417T | T478K | | | Δ211 | | G339D |
|  | T716I | | P681H | | N501Y | | | E484K | | | E484K | D614G | | | L212I | | S371F |
|  | S982A | | T716I | | D614G | | | N501Y | | | N501Y | P681R | | | ins214EPE | | S373P |
|  | D1118H | | S982A | | A701V | | | D614G | | | D614G | D950N | | | G339D | | S375F |
|  |  | | D1118H | |  | | | A701V | | | H655Y |  | | | S371L | | T376A |
|  |  | |  | |  | | |  | | | T1027I |  | | | S373P | | D405N |
|  |  | |  | |  | | |  | | |  |  | | | S375F | | R408S |
| **VOI* pseudovirus** | | | | | | | | | | | |  | | | K417N | | K417N |
| **Epsilon** | **Zeta** | | **Iota** | | **Kappa** | | | **Lambda** | | | **Mu** |  | | | N440K | | N440K |
| B.1.429 | P.2 | | B.1.526 | | B.1.617.1 | | | C.37 | | | B.1.621 |  | | | G446S | | S477N |
| S13I | E484K | | L5F | | T95I | | | G75V | | | Δ69-70 |  | | | S477N | | T478K |
| W152C | D614G | | T95I | | G142D | | | T76I | | | T95I |  | | | T478K | | E484A |
| L452R | V1176F | | D253G | | E154K | | | Δ246-252 | | | Y144S |  | | | E484A | | Q493R |
| D614G |  | | E484K | | L452R | | | D253N | | | Y145N |  | | | Q493K | | Q498R |
|  |  | | D614G | | E484Q | | | L452Q | | | R346K |  | | | G496S | | N501Y |
|  |  | | A701V | | D614G | | | F490S | | | E484K |  | | | Q498R | | Y505H |
|  |  | |  | | P681R | | | D614G | | | N501Y |  | | | N501Y | | D614G |
|  |  | |  | | Q1071H | | | T859N | | | A570D |  | | | Y505H | | H655Y |
|  |  | |  | |  | | |  | | | D614G |  | | | T547K | | N679K |
|  |  | |  | |  | | |  | | | P681H |  | | | D614G | | P681H |
|  |  | |  | |  | | |  | | | T716I |  | | | H655Y | | N764K |
|  |  | |  | |  | | |  | | | D950N |  | | | N679K | | D796Y |
|  |  | |  | |  | | |  | | | S982A |  | | | P681H | | Q954H |
|  |  | |  | |  | | |  | | |  |  | | | N764K | | N969K |
| ^#^VOC, variant of concern; *VOI, variant of interest | | | | | | | | | | | |  | | | D796Y | |  |
|  |  | |  | |  | | |  | | |  |  | | | N856K | |  |
|  |  | |  | |  | | |  | | |  |  | | | Q954H | |  |
|  |  | |  | |  | | |  | | |  |  | | | N969K | |  |
|  |  | |  | |  | | |  | | |  |  | | | L981F | |  |
