# Supplementary material for: Antibody responses against SARS-CoV-2 variants induced by four different SARS-CoV-2 vaccines in health care workers in the Netherlands: A prospective cohort study
Source: PLoS Med. 2022 May 17;19(5):e1003991. doi: 10.1371/journal.pmed.1003991 (PMC9113667; doi:10.1371/journal.pmed.1003991)
Supplement: S2 Table — (DOCX) [file pmed.1003991.s006.docx]

**Table S2: Reported vaccine efficacy.**

| Vaccine | VOC | VE | Reference |
| --- | --- | --- | --- |
| BNT162b | WT | 96% | [1] |
| BNT162b | WT | 95% | [2] |
| BNT162b | WT | 91% | [3] |
| BNT162b | WT | 92% | [4] |
| BNT162b | Alpha | 68% | [5] |
| BNT162b | Alpha | 89% | [6] |
| BNT162b | Alpha | 98% | [7] |
| BNT162b | Alpha | 95% | [8] |
| BNT162b | Alpha | 97% | [9] |
| BNT162b | Alpha | 87% | [10] |
| BNT162b | Alpha | 94% | [11] |
| BNT162b | Alpha | 82% | [12] |
| BNT162b | Alpha | 90% | [13] |
| BNT162b | Alpha | 97% | [14] |
| BNT162b | Alpha | 90% | [15] |
| BNT162b | Alpha | 88% | [4] |
| BNT162b | Alpha | 92% | [16] |
| BNT162b | Alpha | 73% | [16] |
| BNT162b | Beta | 75% | [15] |
| BNT162b | Beta | 86% | [4] |
| BNT162b | Gamma | 90% | [4] |
| BNT162b | Delta | 90% | [17] |
| BNT162b | Delta | 87% | [10] |
| BNT162b | Delta | 93% | [18] |
| BNT162b | Delta | 91% | [19] |
| BNT162b | Delta | 93% | [20] |
| BNT162b | Delta | 92% | [21] |
| BNT162b | Delta | 83% | [8] |
| BNT162b | Delta | 44% | [22] |
| BNT162b | Delta | 88% | [11] |
| BNT162b | Delta | 92% | [4] |
| BNT162b | Omicron | 62% | [23] |
| BNT162b | Omicron | 71% | [18] |
| BNT162b | Omicron | 66% | [8] |
| mRNA-1273 | WT | 94% | [24] |
| mRNA-1273 | WT | 94% | [3] |
| mRNA-1273 | WT | 98% | [4] |
| mRNA-1273 | Alpha | 96% | [6] |
| mRNA-1273 | Alpha | 92% | [4] |
| mRNA-1273 | Delta | 98% | [10] |
| mRNA-1273 | Delta | 95% | [19] |
| mRNA-1273 | Delta | 96% | [21] |
| mRNA-1273 | Delta | 95% | [8] |
| mRNA-1273 | Delta | 74% | [22] |
| mRNA-1273 | Delta | 94% | [4] |
| mRNA-1273 | Omicron | 45% | [23] |
| mRNA-1273 | Omicron | 76% | [19] |
| AZD1222 | WT | 62% | [25] |
| AZD1222 | Alpha | 82% | [8] |
| AZD1222 | Alpha | 78% | [26] |
| AZD1222 | Alpha | 75% | [11] |
| AZD1222 | Alpha | 89% | [13] |
| AZD1222 | Alpha | 87% | [4] |
| AZD1222 | Delta | 72% | [10] |
| AZD1222 | Delta | 83% | [19] |
| AZD1222 | Delta | 68% | [21] |
| AZD1222 | Delta | 64% | [8] |
| AZD1222 | Delta | 67% | [11] |
| AZD1222 | Delta | 88% | [4] |
| AZD1222 | Delta | 79% | [16] |
| AZD1222 | Delta | 60% | [16] |
| AZD1222 | Omicron | 50% | [19] |
| Ad26.COV2.S | WT | 67% | [27] |
| Ad26.COV2.S | Alpha | 77% | [12] |
| Ad26.COV2.S | Beta | 64% | [27] |
| Ad26.COV2.S | Gamma | 51% | [28] |
| Ad26.COV2.S | Delta | 50% | [10] |
| Ad26.COV2.S | Delta | 42% | [12] |

1. Thomas SJ, Moreira ED, Kitchin N, Absalon J, Gurtman A, Lockhart S, et al. Safety and Efficacy of the BNT162b2 mRNA Covid-19 Vaccine through 6 Months. N Engl J Med. 2021. doi:10.1056/NEJMoa2110345

2. Polack FP, Thomas SJ, Kitchin N, Absalon J, Gurtman A, Lockhart S, et al. Safety and Efficacy of the BNT162b2 mRNA Covid-19 Vaccine. N Engl J Med. 2020;383: 2603–2615. doi:10.1056/NEJMoa2034577

3. Chung H, He S, Nasreen S, Sundaram ME, Buchan SA, Wilson SE, et al. Effectiveness of BNT162b2 and mRNA-1273 covid-19 vaccines against symptomatic SARS-CoV-2 infection and severe covid-19 outcomes in Ontario, Canada: test negative design study. BMJ. 2021; n1943. doi:10.1136/bmj.n1943

4. Nasreen S, Chung H, He S, Brown KA, Gubbay JB, Buchan SA, et al. Effectiveness of COVID-19 vaccines against symptomatic SARS-CoV-2 infection and severe outcomes with variants of concern in Ontario. Nat Microbiol. 2022;7: 379–385. doi:10.1038/s41564-021-01053-0

5. Meyer ED, Sandfort M, Bender J, Matysiak-Klose D, Dörre A, Bojara G, et al. Two doses of the mRNA BNT162b2 vaccine reduce severe outcomes, viral load and secondary attack rate: evidence from a SARS-CoV-2 Alpha outbreak in a nursing home in Germany, January-March 2021. medRxiv. 2021; 2021.09.13.21262519. doi:10.1101/2021.09.13.21262519

6. Pilishvili T, Gierke R, Fleming-Dutra KE, Farrar JL, Mohr NM, Talan DA, et al. Effectiveness of mRNA Covid-19 Vaccine among U.S. Health Care Personnel. N Engl J Med. 2021;385: e90. doi:10.1056/NEJMoa2106599

7. Glatman-Freedman A, Bromberg M, Dichtiar R, Hershkovitz Y, Keinan-Boker L, Kissling E, et al. Neutralization of SARS-CoV-2 Omicron by BNT162b2 mRNA vaccine–elicited human sera. Bard JD, editor. N Engl J Med. 2021;385: 379–385. doi:10.1126/science.abn7591

8. Andrews N, Tessier E, Stowe J, Gower C, Kirsebom F, Simmons R, et al. Duration of Protection against Mild and Severe Disease by Covid-19 Vaccines. N Engl J Med. 2022;386: 340–350. doi:10.1056/NEJMoa2115481

9. Katz MA, Harlev EB, Chazan B, Chowers M, Greenberg D, Peretz A, et al. Early effectiveness of BNT162b2 Covid-19 vaccine in preventing SARS-CoV-2 infection in healthcare personnel in six Israeli hospitals (CoVEHPI). Vaccine. 2022;40: 512–520. doi:10.1016/j.vaccine.2021.11.092

10. Kissling E, Hooiveld M, Sandonis Martín V, Martínez-Baz I, William N, Vilcu A-M, et al. Vaccine effectiveness against symptomatic SARS-CoV-2 infection in adults aged 65 years and older in primary care: I-MOVE-COVID-19 project, Europe, December 2020 to May 2021. Eurosurveillance. 2021;26. doi:10.2807/1560-7917.ES.2021.26.29.2100670

11. Lopez Bernal J, Andrews N, Gower C, Gallagher E, Simmons R, Thelwall S, et al. Effectiveness of Covid-19 Vaccines against the B.1.617.2 (Delta) Variant. N Engl J Med. 2021;385: 585–594. doi:10.1056/NEJMoa2108891

12. Martínez-Baz I, Miqueleiz A, Casado I, Navascués A, Trobajo-Sanmartín C, Burgui C, et al. Effectiveness of COVID-19 vaccines in preventing SARS-CoV-2 infection and hospitalisation, Navarre, Spain, January to April 2021. Eurosurveillance. 2021;26. doi:10.2807/1560-7917.ES.2021.26.21.2100438

13. England PH. No Title. Available: https://assets.publishing.service.gov.uk/government/uploads/system/uploads/attachment_data/file/990089/Vaccine_surveillance_report_-_week_20.pdf

14. Angel Y, Spitzer A, Henig O, Saiag E, Sprecher E, Padova H, et al. Association Between Vaccination With BNT162b2 and Incidence of Symptomatic and Asymptomatic SARS-CoV-2 Infections Among Health Care Workers. JAMA. 2021;325: 2457. doi:10.1001/jama.2021.7152

15. Abu-Raddad LJ, Chemaitelly H, Butt AA. Effectiveness of the BNT162b2 Covid-19 Vaccine against the B.1.1.7 and B.1.351 Variants. N Engl J Med. 2021;385: 187–189. doi:10.1056/NEJMc2104974

16. Sheikh A, McMenamin J, Taylor B, Robertson C. SARS-CoV-2 Delta VOC in Scotland: demographics, risk of hospital admission, and vaccine effectiveness. Lancet. 2021;397: 2461–2462. doi:10.1016/S0140-6736(21)01358-1

17. Prunas O, Weinberger DM, Pitzer VE, Gazit S, Patalon T. Waning Effectiveness of the BNT162b2 Vaccine Against Infection in Adolescents. medRxiv. 2022; 2022.01.04.22268776. doi:10.1101/2022.01.04.22268776

18. Powell AA, Kirsebom F, Stowe J, McOwat K, Saliba V, Ramsay ME, et al. Adolescent vaccination with BNT162b2 (Comirnaty, Pfizer-BioNTech) vaccine and effectiveness against COVID-19: national test-negative case-control study, England. medRxiv. 2022; 2021.12.10.21267408. doi:10.1101/2021.12.10.21267408

19. Andrews N, Stowe J, Kirsebom F, Toffa S, Rickeard T, Gallagher E, et al. Covid-19 Vaccine Effectiveness against the Omicron (B.1.1.529) Variant. N Engl J Med. 2022. doi:10.1056/NEJMoa2119451

20. Reis BY, Barda N, Leshchinsky M, Kepten E, Hernán MA, Lipsitch M, et al. Effectiveness of BNT162b2 Vaccine against Delta Variant in Adolescents. N Engl J Med. 2021;385: 2101–2103. doi:10.1056/NEJMc2114290

21. Nordström P, Ballin M, Nordström A. Risk of infection, hospitalisation, and death up to 9 months after a second dose of COVID-19 vaccine: a retrospective, total population cohort study in Sweden. Lancet. 2022;399: 814–823. doi:10.1016/S0140-6736(22)00089-7

22. Tang P, Hasan MR, Chemaitelly H, Yassine HM, Benslimane FM, Al Khatib HA, et al. BNT162b2 and mRNA-1273 COVID-19 vaccine effectiveness against the SARS-CoV-2 Delta variant in Qatar. Nat Med. 2021;27: 2136–2143. doi:10.1038/s41591-021-01583-4

23. Chemaitelly H, Ayoub HH, AlMukdad S, Tang P, Hasan MR, Yassine HM, et al. Duration of protection of BNT162b2 and mRNA-1273 COVID-19 vaccines against symptomatic SARS-CoV-2 Omicron infection in Qatar. medRxiv. 2022; 2022.02.07.22270568. doi:10.1101/2022.02.07.22270568

24. Baden LR, El Sahly HM, Essink B, Kotloff K, Frey S, Novak R, et al. Efficacy and Safety of the mRNA-1273 SARS-CoV-2 Vaccine. N Engl J Med. 2021;384: 403–416. doi:10.1056/NEJMoa2035389

25. Voysey M, Clemens SAC, Madhi SA, Weckx LY, Folegatti PM, Aley PK, et al. Safety and efficacy of the ChAdOx1 nCoV-19 vaccine (AZD1222) against SARS-CoV-2: an interim analysis of four randomised controlled trials in Brazil, South Africa, and the UK. Lancet. 2021;397: 99–111. doi:10.1016/S0140-6736(20)32661-1

26. Hitchings MDT, Ranzani OT, Dorion M, D’Agostini TL, de Paula RC, de Paula OFP, et al. Effectiveness of ChAdOx1 vaccine in older adults during SARS-CoV-2 Gamma variant circulation in São Paulo. Nat Commun. 2021;12: 6220. doi:10.1038/s41467-021-26459-6

27. Sadoff J, Le Gars M, Shukarev G, Heerwegh D, Truyers C, de Groot AM, et al. Interim Results of a Phase 1–2a Trial of Ad26.COV2.S Covid-19 Vaccine. N Engl J Med. 2021;384: 1824–1835. doi:10.1056/NEJMoa2034201

28. Ranzani OT, dos Santos Leite R, Castilho LD, Maymone Gonçalves CC, Resende G, de Melo RL, et al. Vaccine effectiveness of Ad26.COV2.S against symptomatic COVID-19 and clinical outcomes in Brazil: a test-negative study design. medRxiv. 2021; 2021.10.15.21265006. doi:10.1101/2021.10.15.21265006
