# Supplementary material for: Antibody responses against SARS-CoV-2 variants induced by four different SARS-CoV-2 vaccines in health care workers in the Netherlands: A prospective cohort study
Source: PLoS Med. 2022 May 17;19(5):e1003991. doi: 10.1371/journal.pmed.1003991 (PMC9113667; doi:10.1371/journal.pmed.1003991)
Supplement: S3 Table — (DOCX) [file pmed.1003991.s007.docx]

**Table S3: Number of participants included in the binding or neutralization assay per time point per vaccine group.**

|  |  | **Binding** | **Neutralization** |
| --- | --- | --- | --- |
| BNT162b2 | pre-vac | 50 | 0 |
|  | post-V1 | 46 | 45 |
|  | post-V2 | 50 | 50 |
|  | +9m V2 | 42 | 36 |
|  | post-V3 | 37 | 34 |
| mRNA-1273 | pre-vac | 25 | 0 |
|  | post-V1 | 40 | 30 |
|  | post-V2 | 39 | 29 |
|  | +6m V2 | 31 | 24 |
|  | post-V3 | 28 | 20 |
| AZD1222 | pre-vac | 19 | 0 |
|  | post-V1 | 42 | 34 |
|  | post-V2 | 34 | 30 |
|  | +6m V2 | 32 | 29 |
|  | post-V3 | 26 | 23 |
| Ad26.COV2.S | pre-vac | 7 | 0 |
|  | post-V1 | 13 | 13 |
|  | +2m V1 | 13 | 13 |
|  | +7m V1 | 19 | 17 |
|  | post-V2* | 18 | 16 |
