# Supplementary material for: Antibody responses against SARS-CoV-2 variants induced by four different SARS-CoV-2 vaccines in health care workers in the Netherlands: A prospective cohort study
Source: PLoS Med. 2022 May 17;19(5):e1003991. doi: 10.1371/journal.pmed.1003991 (PMC9113667; doi:10.1371/journal.pmed.1003991)
Supplement: S4 Table — (DOCX) [file pmed.1003991.s008.docx]

**Table S4: Uni- and multivariable linear regression analysis and mixed model.**

| **Uni- and multivariable linear regression analysis; Geometric mean titers of antibody binding against Spike protein (wild-type), BAU/ml (95% CI)** | | | | | | | | | | | | | | |
| --- | --- | --- | --- | --- | --- | --- | --- | --- | --- | --- | --- | --- | --- | --- |
|  | **Post 1 vaccination** | | **Post complete initial vaccination±** | | | **Pre-booster** | |  | | **Post-booster** | |  | |  |
|  | **Univariable** | **Multivariable¥** | **Univariable** | **Multivariable¥** | | **Univariable** | | **Multivariable¶** | | **Univariable** | | **Multivariable¥** | |  |
| BNT162b2 | 417 (324-536) | 394 (280-554) | 2967 (2211-3981) | 3077 (2179-4345) | | 192 (138-268) | | 340 (120-963) | | 2840 (2321-3475) | | 2756 (2071-3668) | |  |
| mRNA-1273 | 875 (666-1150)▫▫▫ | 781 (512-1191)▫▫▫ | 3461 (2470-4850) | 3839 (2496-5905) | | 595 (404-876)▫▫▫ | | 386 (211-705) | | 3138 (2488-3958) | | 2993 (2097-4271) | |  |
| AZD1222 | 79 (61-103)*** | 95 (60-150)*** | 219 (153-313)*** | 131 (82-210)*** | | 37 (25-54)*** | | 27 (13-55)*** | | 1487 (1169-1892)*** | | 1480 (997-2196)▫▫ | |  |
| Ad26.COV2.S | 169 (105-271)▪▪ | 169 (96-296)◦◦ | 169 (95-300)*** | 147 (80-272)*** | | 107 (65-175)** | | 90 (49-165)* | | 1541 (1153-2058)*** | | 1484 (1029-2140)*** | |  |
|  |  |  | **Post SARS-CoV-2 infection (4-6 weeks)** | | |  | |  | |  | |  | |  |
| Convalescent# | - | - | 728 (565-939)◦◦◦ | 637 (473-857)◦◦◦ | | - | | - | | - | | - | |  |
| * significantly different from BNT162b2 (p<0.05), mRNA-1273 (p<0.001) and AZD1222 (p<0.01) | | | | | | |  | |  | |  | |  |  |
| ** significantly different from mRNA-1273 (p<0.001) and AZD1222 (p=0.001) | | | | |  | |  | |  | |  | |  |  |
| *** significantly different from both mRNA vaccines (p<0.001) | | | | |  | |  | |  | |  | |  |  |
| ▫▫ significantly different from both mRNA vaccines (p<0.01) | | | |  | |  | |  | |  | |  | |  |
| ▫▫▫ significantly different from BNT162b2 (p<0.001) | | |  |  | |  | |  | |  | |  | |  |
| ▪▪ significantly different from AZD1222 and BNT162b2 (p<0.01) and mRNA-1273 (p<0.001) | | | | | | |  | |  | |  | |  |  |
| ◦◦ significantly different from BNT162b2 (p<0.01) and mRNA-1273 (p<0.001) | | | | |  | |  | |  | |  | |  |  |
| ◦◦◦ significantly different from both mRNA and both vector vaccines (p<0.001) | | | | |  | |  | |  | |  | |  |  |
| All significance statements refer to within column comparisons | | | | |  | |  | |  | |  | |  |  |
|  |  |  |  |  | |  | |  | |  | |  | |  |
| ± complete vaccination is 2 doses of BNT162b2, mRNA-1273 or AZD1222 of 1 dose of Ad26.COV2.S, for Ad26.COV2.S the same time point as post 1 vaccination | | | | | | | | | | | | |  |  |
| ¥ corrected for age and sex, geometric means and %95 CI represent median age of the cohort (47 years ) and female gender | | | | | | | | |  | |  | |  |  |
| ¶ corrected for age, sex and time between full vaccination and pre-booster serum sampling, geometric means and %95 CI represent median age of the cohort (47 years ), female gender and median time between full vaccination and pre-booster serum sampling (28 weeks) | | | | | | | | | | | | | |  |
| # participants of COSCA cohort | |  |  |  | |  | |  | |  | |  | |  |

| **Uni- and multivariable linear regression analysis; Geometric mean titers of antibody neutralization against D614G, IC50 in IU/ml (95% CI)** | | | | | | | | | | | |
| --- | --- | --- | --- | --- | --- | --- | --- | --- | --- | --- | --- |
|  | **Post 1 vaccination** | | **Post complete initial vaccination±** | | | **Pre-booster** | | | | **Post-booster** |  |
|  | **Univariable** | **Multivariable¥** | **Univariable** | **Multivariable** | | **Univariable** | | **Multivariable¶** | **Univariable** | | **Multivariable¥** |
| BNT162b2 | 16 (12-20) | 15 (10-20) | 197 (149-260) | 214 (153-299) | | 24 (18-33) | | 42 (15-113) | 1154 (881-1511) | | 1192 (817-1738) |
| mRNA-1273 | 32 (24-42)** | 28 (18-43)* | 313 (218-448)* | 358 (231-556)* | | 117 (79-172)▫▫▫ | | 92 (53-160) | 1106 (778-1572) | | 1160 (717-1878) |
| AZD1222 | 13 (10-18)▪▪▪ | 13 (8-22)▪▪ | 26 (18-37)*** | 18 (11-30)*** | | 8 (6-12)*** | | 10 (5-19)▪ | 445 (321-617)*** | | 379 (221-650)*** |
| Ad26.COV2.S | 14 (9-22)▪▪ | 13 (8-23)▪▪ | 14 (8-25)*** | 14 (8-25)*** | | 21 (13-33)▫▫ | | 20 (11-35)▪▪▪ | 700 (473-1037)* | | 735 (445-1213)◦ |
|  |  |  | **Post SARS-CoV-2 infection (4-6 weeks)** | | |  | |  |  | |  |
| Convalescent# | - | - | 175 (138-223)◦◦ | 164 (123-219)◦◦◦ | | - | | - | - | | - |
| * significantly different from BNT162b2 (p<0.05) | | |  |  | |  | |  |  | |  |
| ** significantly different from BNT162b2 (p<0.01) | | |  |  | |  | |  |  | |  |
| *** significantly different from both mRNA vaccines (p<0.001) | | | | |  | |  |  |  | |  |
| ▫▫ significantly different from mRNA-1273 (p<0.001) and AZD1222 (p<0.01) | | | | |  | |  |  |  | |  |
| ▫▫▫ significantly different from BNT162b2 (p<0.001) | | |  |  | |  | |  |  | |  |
| ▪ significantly different from BNT162b2 (p<0.05) and mRNA-1273 (p<0.001) | | | | |  | |  |  |  | |  |
| ▪▪ significantly different from mRNA-1273 (p<0.01) | | |  |  | |  | |  |  | |  |
| ▪▪▪ significantly different from mRNA-1273 (p<0.001) | | |  |  | |  | |  |  | |  |
| ◦ significantly different from BNT162b2 and AZD1222 (p<0.05) | | | | |  | |  |  |  | |  |
| ◦◦ significantly different from mRNA-1273 (p<0.01) and both vector vaccines (p<0.001) | | | | | | |  |  |  | |  |
| ◦◦◦ significantly different from mRNA-1273 (p<0.001) and both vector vaccines (p<0.001) | | | | | | |  |  |  | |  |
| All significance statements refer to within column comparisons | | | | |  | |  |  |  | |  |
|  |  |  |  |  | |  | |  |  | |  |
| ± complete vaccination is 2 doses of BNT162b2, mRNA-1273 or AZD1222 of 1 dose of Ad26.COV2.S | | | | | | |  |  |  | |  |
| ¥ corrected for age and sex, geometric mean titers and %95 CI represent median age of the cohort (47 years ) and female gender | | | | | | | | |  | |  |
| ¶ corrected for age, sex and time between full vaccination and pre-booster serum sampling, geometric mean titers and %95 CI represent median age of the cohort (47 years ), female gender and median time between full vaccination and pre-booster serum sampling (28 weeks) | | | | | | | | | | | |
| # participants of COSCA cohort | |  |  |  | |  | |  |  | |  |

| **Uni- and multivariable linear mixed model; Fold change of binding antibody geometric mean titers against wild-type between measurements** | | | | | | | | | |
| --- | --- | --- | --- | --- | --- | --- | --- | --- | --- |
|  | **From post initial vaccination to pre-booster** | | **From post initial vaccination to post-booster** | | | | **From pre-booster to post-booster** | | |
|  | **Univariable** | **Multivariable¶** | **Univariable** | | **Multivariable¥** | | **Univariable** | | **Multivariable¥** |
| BNT162b2 | 0.06 (0.04-0.10) | 0.14 (0.04-0.41) | 0.97 (0.60-1.57) | | 0.98 (0.61-1.58) | | 14.95 (9.09-24.56) | | 14.94 (9.08-24.52) |
| mRNA-1273 | 0.18 (0.10-0.30)** | 0.16 (0.09-0.27) | 0.92 (0.53-1.60) | | 0.94 (0.54-1.62) | | 5.28 (2.97-9.37)▪▪ | | 5.29 (2.98-9.39)▪▪ |
| AZD1222 | 0.17 (0.10-0.30)** | 0.13 (0.07-0.25) | 6.93 (3.89-12.33)▪▪▪ | | 6.86 (3.85-12.20)▪▪▪ | | 40.38 (22.55-72.27)▪ | | 40.12 (22.39-71.73)▪ |
| Ad26.COV2.S | 0.44 (0.20-0.98)*** | 0.46 (0.20-1.03)* | 6.31 (2.79-14.28)▪▪▪ | | 6.26 (2.76-14.15)▪▪▪ | | 14.42 (6.99-29.73)* | | 14.40 (6.98-29.69)* |
| *** significantly different from fold change of BNT162b2 (p<0.001) | | | |  | |  | |  | |
| ** significantly different from fold change of BNT162b2 (p<0.01) | | | |  | |  | |  | |
| * significantly different from fold change of mRNA-1273 and AZD1222 (p<0.05) | | | | | |  | |  | |
| ▪▪▪ significantly different from fold change of mRNA-1273 and BNT162b2 (p<0.001) | | | | | |  | |  | |
| ▪▪ significantly different from fold change of BNT162b2 (p<0.01) | | | |  | |  | |  | |
| ▪ significantly different from fold change of BNT162b2 (p<0.05) and mRNA-1273 (p<0.001) | | | | | |  | |  | |
| All significance statements refer to within column comparisons | | | |  | |  | |  | |
|  |  |  |  | |  | |  | |  |
| ¥ corrected for age and sex, geometric mean titers and %95 CI represent median age of the cohort (47 years ) and female gender | | | | | | | | | |
| ¶ corrected for age, sex and time between full vaccination and pre-booster serum sampling, geometric mean titers and %95 CI represent median age of the cohort (47 years ), female gender and median time between full vaccination and pre-booster serum sampling (28 weeks) | | | | | | | | | |

| **Uni- and multivariable linear mixed model; Fold change of neutralizing antibody geometric mean titers against D614G between measurements** | | | | | | |
| --- | --- | --- | --- | --- | --- | --- |
|  | **From post initial vaccination to pre-booster** | | **From post initial vaccination to post-booster** | | **From pre-booster to post-booster** | |
|  | **Univariable** | **Multivariable¶** | **Univariable** | **Multivariable¥** | **Univariable** | **Multivariable¥** |
| BNT162b2 | 0.13 (0.10-0.17) | 0.18 (0.08-0.38) | 6.05 (4.54-8.07) | 6.08 (4.55-8.10) | 46.81 (34.45-63.61) | 46.85 (34.50-63.68) |
| mRNA-1273 | 0.38 (0.27-0.54)*** | 0.37 (0.25-0.53) | 3.69 (2.53-5.37)* | 3.73 (2.56-5.43)* | 9.71 (6.60-14.29)*** | 9.74 (6.62-14.34)*** |
| AZD1222 | 0.33 (0.23-0.46)*** | 0.29 (0.19-0.45) | 17.86 (12.42-25.68)▫▫▫ | 17.83 (12.39-25.61)▫▫▫ | 54.71 (38.24-78.25)◦◦◦ | 54.54 (38.11-77.96)◦◦◦ |
| Ad26.COV2.S | 1.22 (0.73-2.06)▪▪▪ | 1.24 (0.74-2.10)▪▪▪ | 41.33 (24.25-70.44)▪ | 41.03 (24.04-69.83)▪ | 33.81 (21.73-52.61)◦◦◦ | 33.79 (21.72-52.56)◦◦◦ |
| *** significantly different from fold change of BNT162b2 (p<0.001) | | | |  |  |  |
| ** significantly different from fold change of BNT162b2 (p<0.001) and Ad.COV2.S (p<0.01) | | | | |  |  |
| * significantly different from fold change of BNT162b2 (p<0.05) | | | |  |  |  |
| ▫▫▫ significantly different from fold change of mRNA-1273 and BNT162b2 (p<0.001) | | | | |  |  |
| ▪▪▪ significantly different from fold change of mRNA-1273, BNT162b2 and AZD1222 (p<0.001) | | | | |  |  |
| ▪▪ significantly different from fold change of mRNA-1273 and AZD1222 (p<0.01) and BNT162b2 (p<0.001) | | | | |  |  |
| ▪ significantly different from fold change of AZD1222 (p<0.05) and BNT162b2 and mRNA-1273 (p<0.001) | | | | |  |  |
| ◦◦◦ significantly different from fold change of mRNA-1273 (p<0.001) | | | |  |  |  |
| All significance statements refer to within column comparisons | | | |  |  |  |
|  |  |  |  |  |  |  |
| ¥ corrected for age and sex, geometric mean titers and %95 CI represent median age of the cohort (47 years ) and female gender | | | | | |  |
| ¶ corrected for age, sex and time between full vaccination and pre-booster serum sampling, geometric mean titers and %95 CI represent median age of the cohort (47 years ), female gender and median time between full vaccination and pre-booster serum sampling (28 weeks) | | | | | | |
